# Integrated Multi-Omics Analysis Provides No Evidence for Pathogenicity of *Balantidium Ctenopharyngodoni* in Grass Carp Hindgut

**DOI:** 10.3390/ijms27219425

**Published:** 2026-10-22

**Authors:** Weishan Zhao, Lei Fang, Weitian Zhou, Xialian Bu, Shangong Wu, Hong Zou, Wenxiang Li, Guitang Wang, Ming Li

**Affiliations:** 1 State Key Laboratory of Breeding Biotechnology and Sustainable Aquaculture, Wuhan 430072, China; zws@ihb.ac.cn (W.Z.);; 2 Institute of Hydrobiology, Chinese Academy of Sciences, Wuhan 430072, China; 3 Jiujiang Academy of Agricultural Sciences, Jiujiang 332000, China; 4 Freshwater Fisheries Research Institute of Fujian, Fuzhou 350002, China; 5 Zhejiang Institute of Freshwater Fisheries, Huzhou 313001, China

**Keywords:** *Balantidium ctenopharyngodoni*, intestinal ciliate, integrative analysis, dietary regime, grass carp

## Abstract

*Balantidium ctenopharyngodoni* Chen, 1955 is the only ciliate species known to inhabit the digestive tract of grass carp, where it predominantly resides in the spaces between mucosal folds and within the intestinal contents of the hindgut with a high prevalence. Despite its high prevalence, the pathogenicity remains controversial. The intestinal bacterial microbiota interacts closely with enteric protozoa, and such interactions may alter host–parasite dynamics, with consequences for host susceptibility and overall health. In this study, we conducted a feeding experiment with three different dietary regimes to explore the effects of *B. ctenopharyngodoni* colonization on grass carp, using an integrative approach that combined microbial amplicon sequencing, metabolomics, and intestinal transcriptomics. Our results showed that balantidia colonization was associated with slightly lower body length and body weight, although these differences were not statistically significant. Balantidia colonization was also related to altered microbiota composition and diversity: the relative abundance of Bacteroidetes was more abundant in the balantidia-positive fish with a plant-based diet, while alphaproteobacteria and betaproteobacteria were enriched in the balantidia-positive fish with commercial feed. Although *B. ctenopharyngodoni* moderately perturbed the microbial profiles and metabolic processes of the grass carp hindgut, intestinal transcriptomic analysis did not detect significant transcriptional changes in immune-related genes between balantidia-positive and balantidia-negative groups. Collectively, under the tested dietary and physiological conditions, our findings provide no evidence of overt pathogenicity, further supporting the view that *B. ctenopharyngodoni* acts as a commensal ciliate in the grass carp hindgut.

## 1. Introduction

Grass carp (*Ctenopharyngodon idella*) is one of “four major domestic fishes” in China and is highly valued for aquaculture due to its rapid growth rate [1]. The fish fry feed on zooplankton, the juveniles feed on insects and duckweed, and then they feed exclusively on aquatic plants as adults when the body length reaches about 10 cm [1,2,3]. *Balantidium ctenopharyngodoni* (hereafter referred to as balantidia) is a common intestinal ciliate inhabiting the hindgut of grass carp aged two summers and older (with body length far exceeding 10 cm). Although it has been considered a low-pathogenic or possibly opportunistic parasite that resides in the intestinal content between mucosal folds, it may aggravate the symptoms when its host suffers from enteritis [4,5,6,7,8,9]. Therefore, its pathogenicity remains unclear.

The intestinal tract harbors a complex community of bacteria, fungi and protozoa, which interact with the host in diverse ways, including food digestion, nutrient absorption, and immune regulation [10]. The protist-microbiota interactions are fundamental to understanding the ecological and biological traits of protists, since microbial interactions are pivotal to the intestinal microbiome [11]. Accumulating evidence suggests that the gut microbiota serves as a key mediator of parasite–host relationships, not only through direct contact with the parasite but by modulating the host’s immune response [12,13]. An in vitro cultivation experiment has demonstrated that *B. ctenopharyngodoni* was strongly associated with intestinal bacteria [6,14], and its presence was linked to higher bacterial diversity in the intestinal contents of grass carp [5]. Both commensal and pathogenic intestinal protozoa can cause shifts in the microbial community during colonization, leading to distinct microbiome profiles and disease outcomes. These shifts may help explain the divergent types of mucosal immune responses that develop and whether a protozoan is regarded as a commensal or a pathogen [15]. Pathogenicity may be linked to the overgrowth of specific microbes, whose uncontrolled proliferation can drive protozoa toward a pathogenic state [16,17]. However, many well-studied parasites, such as species of *Entamoeba*, *Giardia*, and *Blastocystis*, are frequently found in asymptomatic hosts, lacking clear evidence of acute pathogenicity. In contrast, higher relative abundances of beneficial bacteria and increased bacterial diversity were often observed in the intestines colonized by these commensal species [11,18,19,20].

Beyond direct microbial interactions, the intestinal microbiota also affects the host immune system through its metabolic products, which participate in regulating immune response of host [10]. Beneficial metabolites can enhance host growth performance, intestinal function and immune status, while also inhibiting disease progression [21]. Consequently, the dynamic interplay among parasites, host microbiota, metabolites, and the host immune system determines the outcome of enteric infections. A broader understanding of these multifaceted interactions is essential to accurately capture the complex host–parasite–microbiota relationships in natural situations [22,23]. Alteration in intestinal microbiota and host physiological status under different environmental conditions may provide insights into the immune responses that regulate the progression of inflammation in parasitic diseases.

In this study, we conducted a comprehensive analysis to investigate the effects of balantidia colonization on the hindgut of grass carp reared under different diets, because its pathogenicity still remains controversial. We sought to investigate whether colonization by balantidia under the tested dietary and physiological conditions is associated with changes in the hindgut microbiota, metabolome, and host transcriptional responses. To this end, microbial amplicon sequencing, untargeted metabolomics, and intestinal transcriptomics were used to characterize microbial composition and diversity, metabolite profiles, and intestinal immune factors, respectively. Through these integrative analyses, we aimed to evaluate the pathogenicity of *B. ctenopharyngodoni* and its comprehensive effects on the host hindgut. This study will enhance our understanding of the commensal role of balantidia and evaluate whether colonization is associated with pathological, immunological, or growth effects in grass carp under different dietary regimes.

## 2. Results

### 2.1. Growth Performance and Infection Intensity

**Figure 1:**
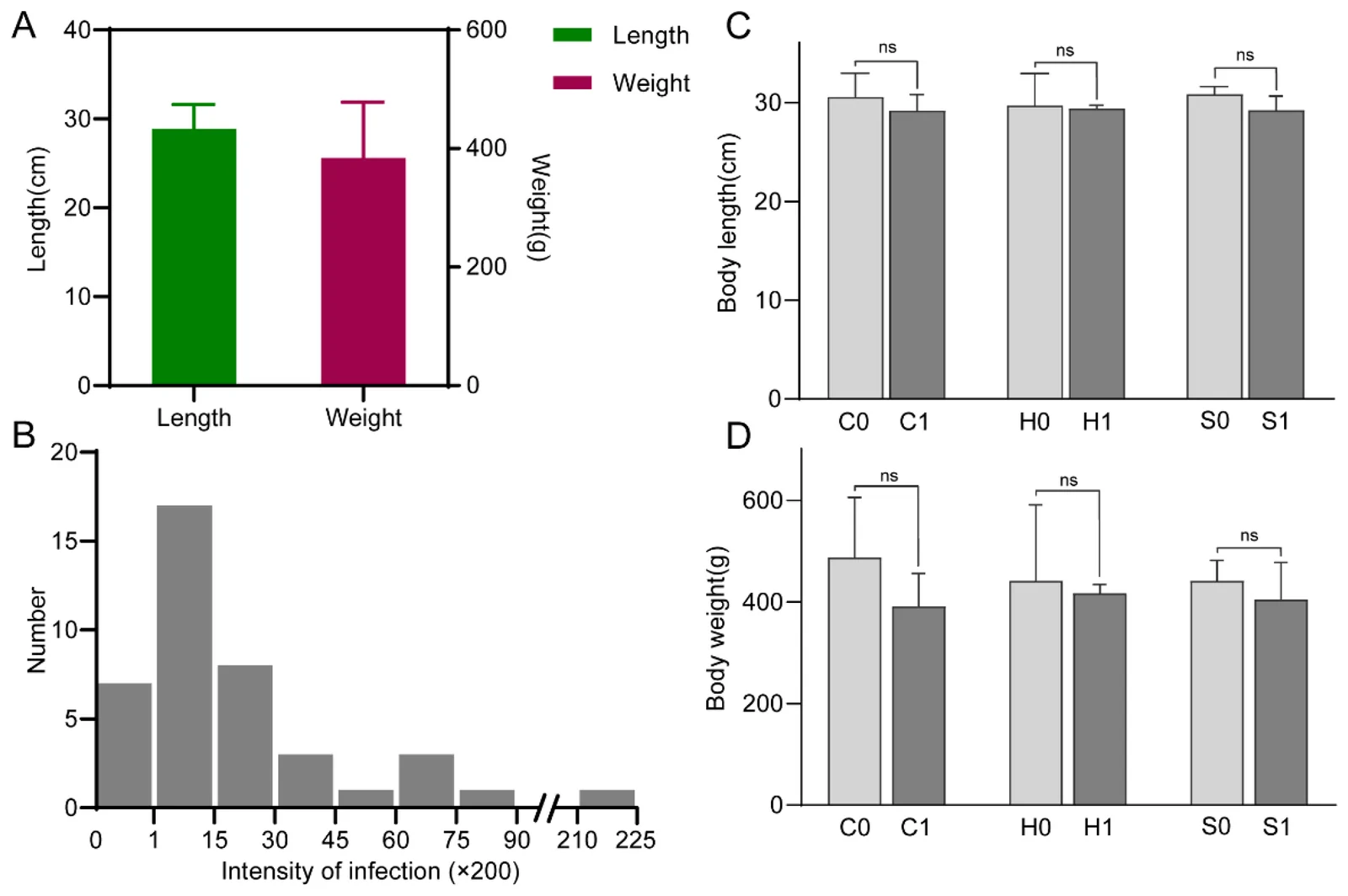
Body length and weight of grass carp and infection intensity of *Balantidium ctenopharyngodoni*. (**A**) Body length and weight of grass carp prior to the rearing experiments. Data are shown as mean ± SD, *n* = 41 fish. (**B**) Frequency distribution of infection intensity of *B. ctenopharyngodoni* before the experiments. (**C**–**D**) Comparison of body length (**C**) and body weight (**D**) between balantidia-negative and balantidia-positive grass carp after the rearing experiment (ns, not significant; *p* > 0.05). Data are shown as mean ± SD, *n* = 5 fish per subgroup. Groups: C, plant-based diet (ryegrass); H, mixed diet (ryegrass + commercial feed); S, commercial feed only. “0” indicates balantidia-negative fish; “1” indicates balantidia-positive fish.

Prior to the experiment, the mean body length and body weight of grass carp were 28.88 ± 2.73 cm and 384.31 ± 94.14 g, respectively (Figure 1A). The frequency distribution of infection intensity in the host population was highly aggregated, with the majority of individuals harboring low balantidia numbers (Figure 1B). Notably, qualitative histological examination revealed no obvious intestinal damage in any of the examined fish, regardless of infection intensity (Figure S1). At the end of the experiment, body length and body weight tended to be slightly lower in balantidia-positive fish than in balantidia-negative fish within each group; however, no statistically significant differences were detected (*p* > 0.05, Figure 1C,D).

### 2.2. Microbial Community Composition and Diversity

**Figure 2:**
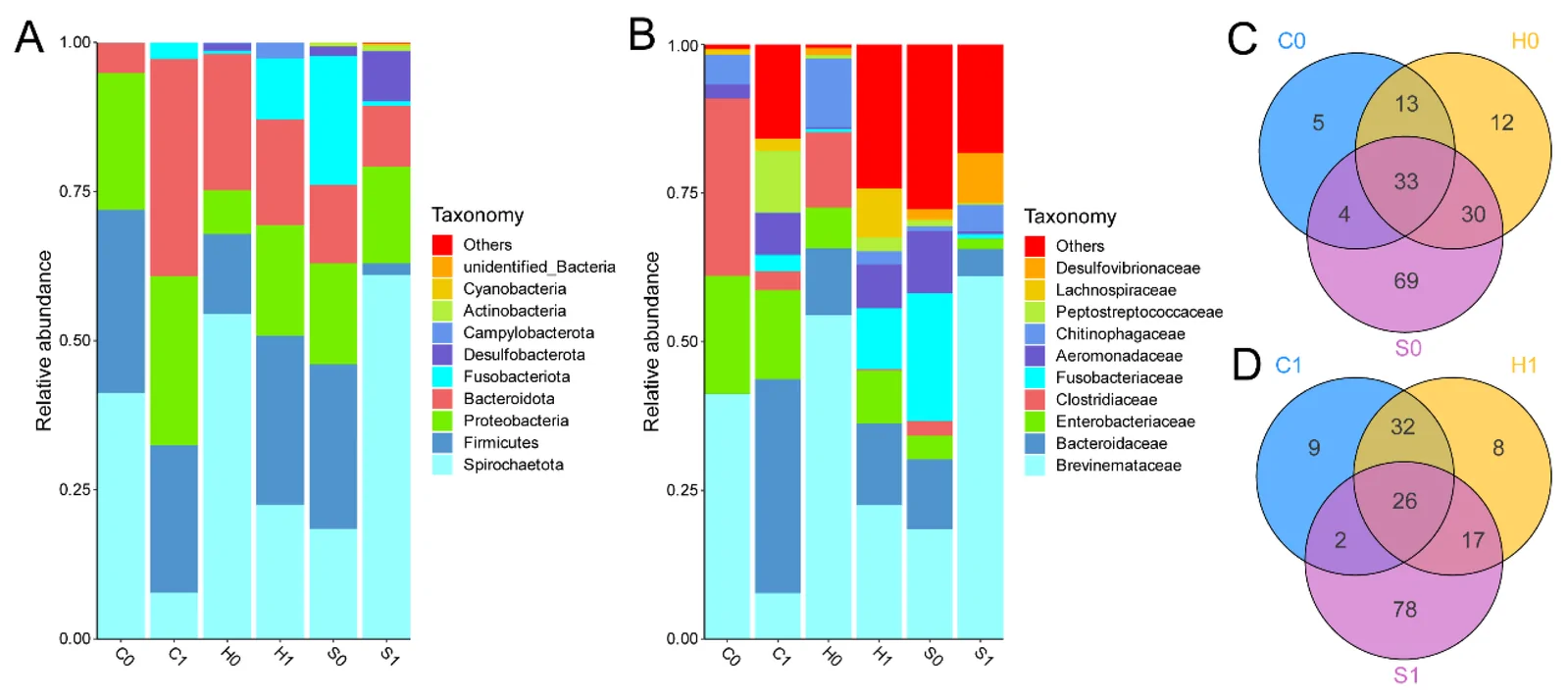
Microbial community composition across experimental groups. (**A**,**B**) Relative abundance of microbial communities at the phylum level (**A**) and the family level (**B**). (**C**,**D**) Venn diagram showing numbers of shared and unique ASVs among the three Balantidium–negative groups (**C**) and the three Balantidium–positive groups (**D**). *n* = 5 per group. Groups: C, plant–based diet (ryegrass); H, mixed diet (ryegrass + commercial feed); S, commercial feed only. “0” indicates balantidia–negative fish; “1” indicates balantidia–positive fish.

A total of 6,419,240 raw reads were obtained from 30 intestinal content samples. After quality filtering, 6,396,622 clean reads were retained, with a mean GC content of 51.27% (Table S1). Based on sequence similarity, bacterial taxa across all samples were assigned to 12 phyla, 15 classes, and 69 families. At the phylum level, Spirochaetota, Firmicutes, Proteobacteria, and Bacteroidota were the predominant taxa across all groups (Figure 2A). In the C group, the relative abundance of Bacteroidota (5.1% to 36.4%), Proteobacteria (22.9% to 28.3%) and Fusobacteriota (0.05% to 2.8%) was higher in group C1, accompanied by a lower relative abundance in Spirochaetota (41.2% to 7.7%) and Firmicutes (30.7% to 24.8%). In the H group, group H1 exhibited a lower relative abundance in Spirochaetota (54.4% to 22.5%) and an expansion of Fusobacteriota (0.6% to 10.3%), Proteobacteria (7.3% to 18.5%), and Firmicutes (13.4% to 28.3%) relative to group H0. In the group S comparison, group S1 was characterized by a higher relative abundance in Spirochaetota (18.5% to 61.0%), whereas Firmicutes (27.6% to 1.9%) and Fusobacteriota (21.6% to 0.7%) were lower.

At the family level (Figure 2B), the C1 vs. C0 comparison revealed an enrichment of Bacteroidaceae (from 0.02% to 36.0%), and a concurrent reduction in Brevinemataceae (41.2% to 7.7%) and Clostridiaceae (29.8% to 3.2%). In the H1 vs. H0 comparison, both Brevinemataceae (54.4% to 22.5%) and Clostridiaceae (12.7% to 0.2%) declined, while Fusobacteriaceae (0.6% to 10.3%) and Aeromonadaceae (0.2% to 7.4%) increased. In the S group, S1 was dominated by a dramatic increase in Brevinemataceae (18.5% to 61.0%), accompanied by reductions in Fusobacteriaceae (21.5% to 0.7%) and Aeromonadaceae (10.4% to 0.5%).

**Figure 3:**
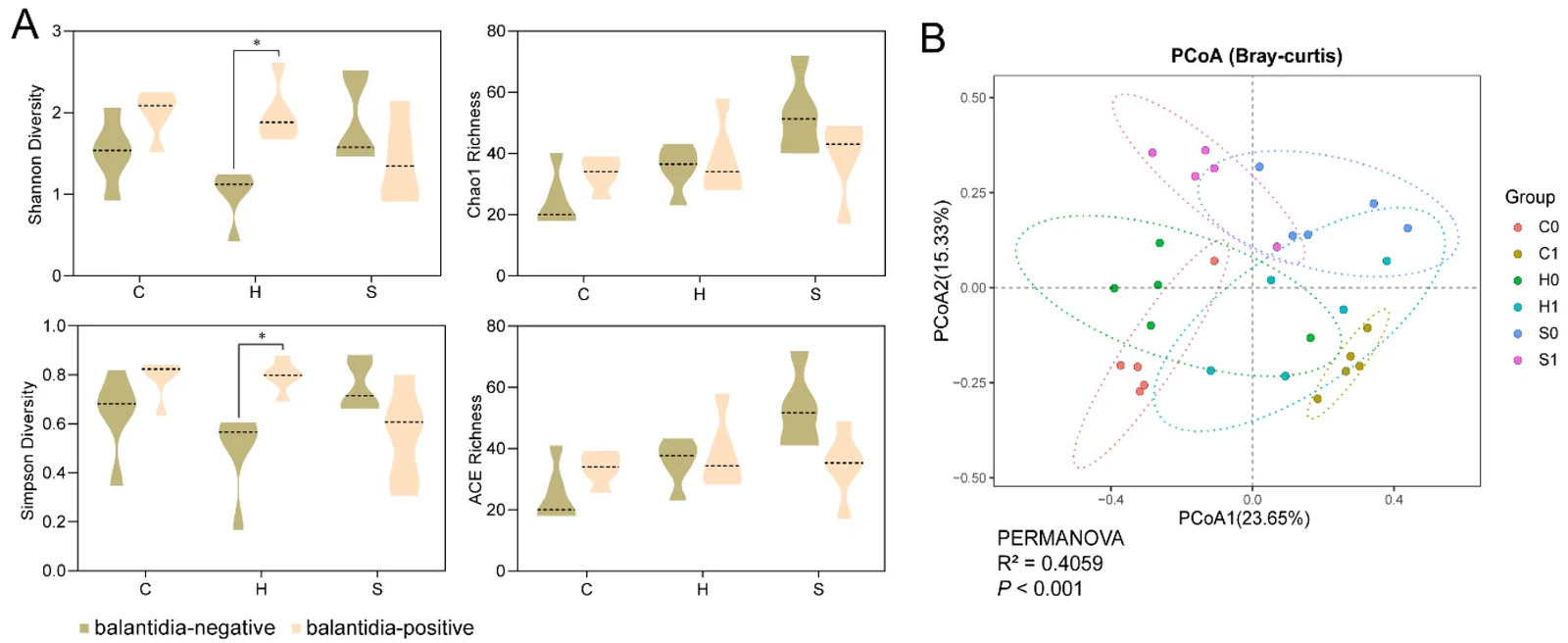
Diversity of microbial community across experimental groups. (**A**) Alpha diversity indices (Shannon, Simpson, Chao1, and ACE) across groups based on colonization status. * indicates significant (q < 0.05, Mann–Whitney U test with FDR-adjusted *p* values). (**B**) Principal coordinate analysis (PCoA) based on Bray–Curtis distance on six groups of microbial communities. Groups: C, plant-based diet (ryegrass); H, mixed diet (ryegrass + commercial feed); S, commercial feed only. “0” indicates balantidia-negative fish; “1” indicates balantidia-positive fish.

The number of shared and unique ASVs was identified across groups. In the balantidia-negative groups, 33 core ASVs were shared by all three groups (Figure 2C), whereas 26 core ASVs were shared in the balantidia-positive groups (Figure 2D). Alpha diversity and richness of the microbial community, measured by Shannon, Simpson, Chao1, and ACE indices, are shown in Figure 3A and Table 1. Two-way ANOVA was used to test the main effects of diet and balantidia status as well as their interaction (Table S2). Diet had a significant effect on Chao1 (*p* = 0.007, q = 0.028) and ACE (*p* = 0.014, q = 0.042) richness, whereas the effect of balantidia was not significant for any index (all q > 0.05). Notably, a significant Diet × Status interaction was detected for Shannon (*p* = 0.002, q = 0.014) and Simpson (*p* = 0.002, q = 0.014) diversity (Table S2). In groups C and H, both mean and median values of Shannon and Simpson diversity indices, as well as Chao1 and ACE richness indices, were higher in the balantidia-positive group than in the balantidia-negative group. In contrast, in group S, these indices were lower in the balantidia-positive group. Within-diet comparisons showed that H1 fish had a significantly higher Shannon (q < 0.05) and Simpson (q < 0.05) diversity than H0 fish (Table 1). In contrast, no statistically significant differences were detected between positive and negative fish in the C group and S group. In addition, no significant differences were detected in any dietary group for Chao1 and ACE indices (all q > 0.05).

Principal coordinate analysis (PCoA) based on Bray–Curtis distances revealed clear separation of microbial communities according to both dietary group (C/H/S) and infection status (Figure 3B). Two-way PERMANOVA on Bray–Curtis distances showed that diet (R^2^ = 0.137, F = 2.96, *p* = 0.001), balantidia status (R^2^ = 0.076, F = 3.27, *p* = 0.008), and their interaction (R^2^ = 0.231, F = 4.97, *p* = 0.001) were all significantly associated with microbial community structure (Table S3). Pairwise PERMANOVA among dietary groups showed that the C and S groups differed significantly (R^2^ = 0.166, q = 0.006), whereas C vs. H (q = 0.054) and H vs. S (q = 0.129) were not significant (Table S4). Within each dietary group, positive and negative fish differed significantly in microbial community structure (C1 vs. C0: R^2^ = 0.481, q = 0.030; H1 vs. H0: R^2^ = 0.203, q = 0.034; S1 vs. S0: R^2^ = 0.389, q = 0.030; Table S5).

**Figure 4:**
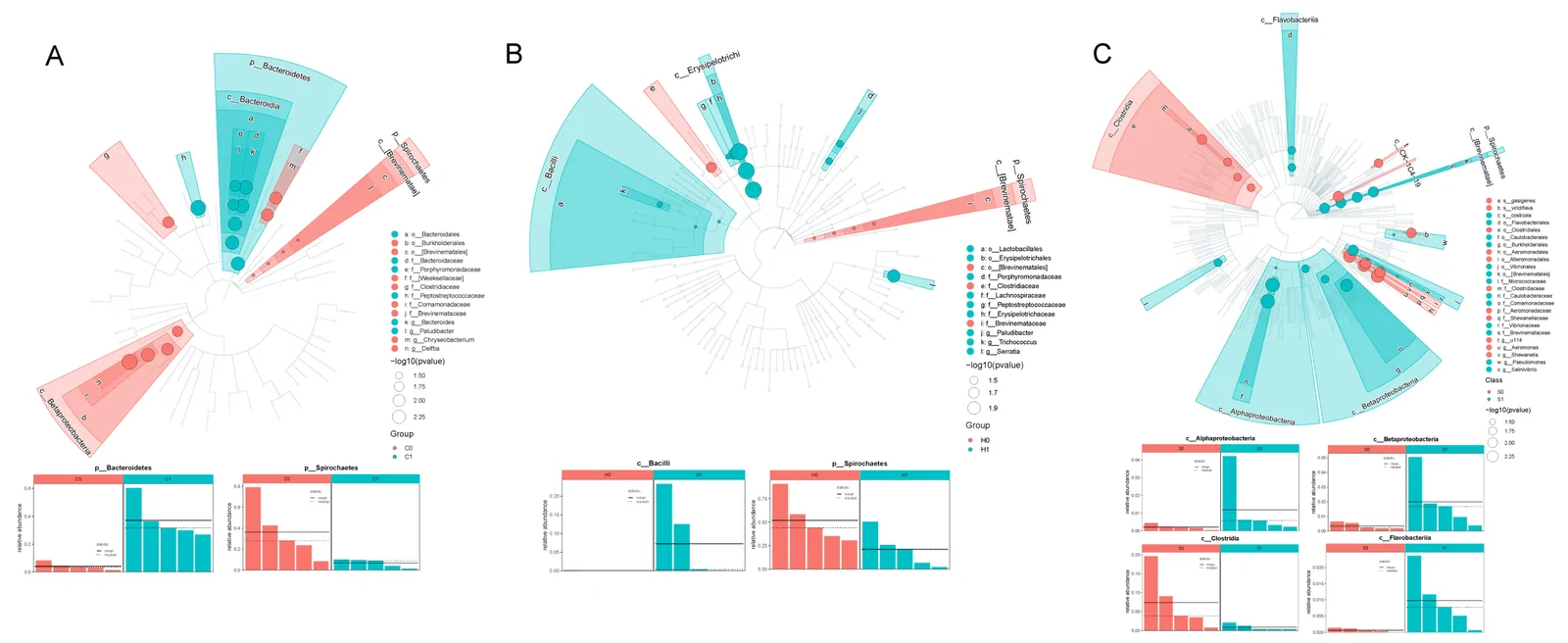
Taxonomic cladogram showing differentially abundant microbial biomarkers between C0 and C1 groups (**A**), H0 and H1 groups (**B**), and S0 and S1 groups (**C**). Groups: C, plant-based diet (ryegrass); H, mixed diet (ryegrass + commercial feed); S, commercial feed only. “0” indicates balantidia-negative fish; “1” indicates balantidia-positive fish.

Biomarker discovery analysis using linear discriminant analysis (LDA) effect size (LEfSe) was performed to identify bacterial taxa significantly enriched in either balantidia-negative or balantidia-positive groups. Bacteroidetes was identified as a key biomarker for the C1 group, while Spirochaetes and Betaproteobacteria were prominent biomarkers for the C0 group (Figure 4A). In the H group comparison, taxa affiliated with the class Bacilli were significantly enriched in the H1 group (Figure 4B). In the S group, the classes Alphaproteobacteria, Betaproteobacteria, and Flavobacteriia were significantly enriched in S1, whereas members of the class Clostridia were characteristic biomarkers of S0 (Figure 4C). In addition, balantidia colonization significantly reduced the Firmicutes/Bacteroidetes (F/B) ratio in the grass carp hindgut regardless of dietary type (*p* < 0.05; Figure S2).

### 2.3. Metabolomics Analysis and Integrated Microbiome-Metabolome Analysis

**Figure 5:**
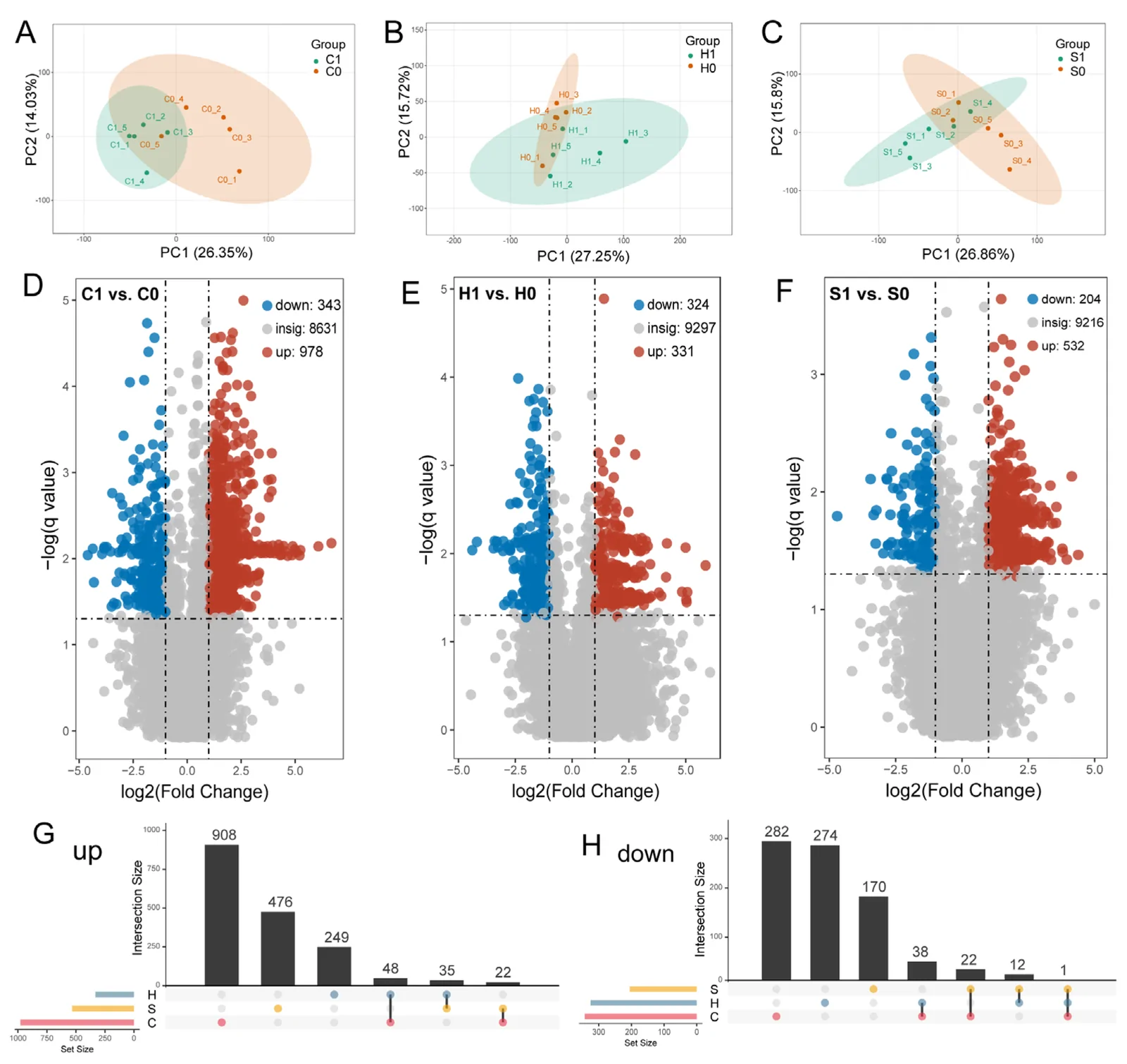
Differential metabolite profiles and KEGG pathway enrichment analysis across groups (*n* = 5 per group). (**A**–**C**) Principal component analysis (PCA) of metabolic profiles: C1 vs. C0 (**A**), H1 vs. H0 (**B**), and S1 vs. S0 (**C**). (**D**–**F**) Volcano plots showing differentially accumulated metabolites (DAMs) between balantidia-positive and balantidia-negative groups: C1 vs. C0 (**D**), H1 vs. H0 (**E**), and S1 vs. S0 (F). (**G**,**H**) Upset diagrams showing upregulated DAMs (**G**) and downregulated DAMs (**H**) among the three balantidia-positive groups. Groups: C, plant-based diet (ryegrass); H, mixed diet (ryegrass + commercial feed); S, commercial feed only. “0” indicates balantidia-negative fish; “1” indicates balantidia-positive fish.

Differences in the intestinal content metabolites between balantidia-negative and balantidia-positive groups were assessed by principal component analysis. The results indicated that balantidia colonization altered the intestinal metabolic profiles in all three dietary groups (Figure 5A–C). For the C group comparison (C1 vs. C0), a total of 1321 differentially accumulated metabolites (DAMs) were significantly altered, of which 978 were up-accumulated and 343 were down-accumulated in the balantidia-positive group (Figure 5D). In the H group (H1 vs. H0), 655 DAMs were identified, with 331 increased and 324 decreased (Figure 5E). For the S group (S1 vs. S0), 736 DAMs were detected, including 532 up-accumulated and 204 down-accumulated metabolites (Figure 5F). Among up-accumulated metabolites, the number of group-specific metabolites was highest in the C group (908), followed by the H and S groups (Figure 5G). Group-specific down-accumulated metabolites were also most abundant in the C group (282), followed by the H and S groups (Figure 5H).

Two-way PERMANOVA on metabolomic profiles showed that diet (R^2^ = 0.260, F = 5.287, *p* = 0.001), balantidia status (R^2^ = 0.064, F = 2.596, *p* = 0.007), and their interaction (R^2^ = 0.085, F = 1.721, *p* = 0.031) were all significantly associated with the overall metabolomic profiles (Table S6). Diet explained the largest proportion of variation (26.0%), followed by the interaction (8.5%) and status (6.4%). Pairwise PERMANOVA among dietary groups showed that all three diets harbored distinct metabolomic profiles (C vs. H: R^2^ = 0.165, q = 0.001; C vs. S: R^2^ = 0.314, q = 0.001; H vs. S: R^2^ = 0.135, q = 0.001; Table S7). Within each dietary group, balantidia-positive and balantidia-negative fish differed significantly in the C group (C1 vs. C0: R^2^ = 0.249, q = 0.039) and the S group (S1 vs. S0: R^2^ = 0.190, q = 0.039), whereas the difference in the H group was not significant (H1 vs. H0: R^2^ = 0.169, q = 0.080; Table S8).

KEGG pathway enrichment analysis was performed to explore the functional implications of the DAMs. In the C1 vs. C0 comparison, the DAMs were associated with many metabolic pathways, including amino acid metabolism, core energy metabolism, and nucleotide metabolism (Figure S3A). In the H group comparison (H1 vs. H0), the significantly enriched pathways were related to biosynthesis of secondary metabolites and lipid metabolism (Figure S3B). For the S1 vs. S0 comparison, arachidonic acid metabolism was the most significantly enriched pathway, and multiple pathways related to steroid hormone metabolism, including steroid hormone biosynthesis and degradation, were also perturbed (Figure S3C).

Correlation network analysis was performed to explore the relationships between significantly altered microbial taxa and differential metabolites in each comparison. In the C group (Figure S4A), key microbial taxa including unidentified Clostridia and Bacteroides were highly connected with numerous metabolites. In the H group (Figure S4B), *Erysipelatoclostridium*, Peptostreptococcaceae and Bacilli were central taxa in the correlation network. In the S group (Figure S4C), Clostridiaceae was the dominant node with the highest degree, showing extensive negative correlations with metabolites.

### 2.4. Differentially Expressed Genes (DEGs) and Verification

**Figure 6:**
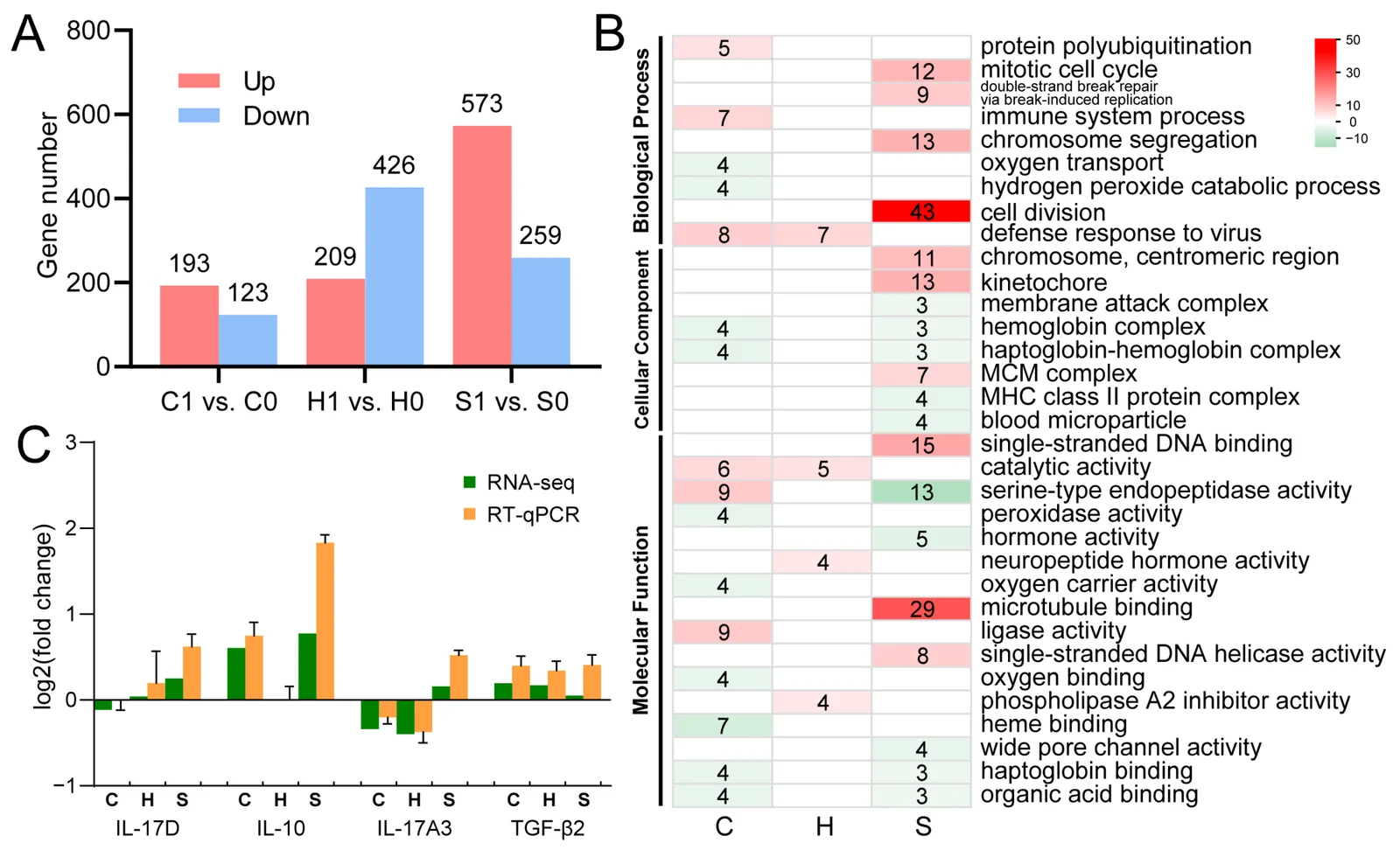
Transcriptomic profiles and qRT-PCR validation of the grass carp hindgut under different dietary regimes. (**A**) Expression profiles and (**B**) functional enrichment analysis of differentially expressed genes (DEGs). (**C**) Validation of RNA-seq results by quantitative real-time PCR (qRT-PCR; *n* = 3 per group). The relative expression levels of genes, represented as log_2_(fold change), are compared with RNA-seq results. Groups: C, plant-based diet (ryegrass); H, mixed diet (ryegrass + commercial feed); S, commercial feed only. “0” indicates balantidia-negative fish; “1” indicates balantidia-positive fish.

To analyze the intestinal transcriptional profiles of balantidia-positive fish under different dietary regimes, transcriptome sequencing was performed on hindgut tissues from both balantidia-negative and balantidia-positive fish. Two-way PERMANOVA on Euclidean distances of the transcriptomic data showed that diet had a significant effect (R^2^ = 0.161, *p* = 0.001), whereas the main effect of balantidia status (R^2^ = 0.032, *p* = 0.131) and the Diet × Status interaction (R^2^ = 0.053, *p* = 0.316) were not significant (Table S9). These results indicate that diet was the dominant factor shaping the overall hindgut transcriptome, while colonization status was not associated with global transcriptional response. In the C1 vs. C0 comparison (Figure 6A and Figure S5A), a total of 316 DEGs were identified, with 193 significantly up-regulated and 123 significantly down-regulated (q < 0.05). In the H1 vs. H0 group (Figure 6A and Figure S5B), 635 DEGs were detected, of which 209 were significantly up-regulated and 426 were significantly down-regulated (q < 0.05). In the S1 vs. S0 comparison (Figure 6A and Figure S5C), 832 DEGs were observed, with 573 significantly up-regulated and 259 significantly down-regulated (q < 0.05). A hierarchical clustering heatmap of all DEGs was constructed to visualize global transcriptional patterns across all groups. Sample clustering distinctly separated all balantidia-positive groups from their balantidia-negative counterparts (Figure S5D).

Gene ontology (GO) enrichment analysis was performed to characterize the functional features of DEGs across the balantidia-positive C, H, and S groups (Figure 6B). In the C group, genes involved in ligase activity, peptidase activity, and defense response to virus were detected as up-regulated. The H group was characterized by up-regulated genes related to defense response to virus and catalytic activity. In the S1 vs. S0 comparison, genes enriched in cell division, microtubule binding and DNA replication-related functions were up-regulated.

We focused on a subset of genes involved in both innate and adaptive immunity that are related to immune responses. No significant alterations in transcription levels were detected for most immune-related genes in the balantidia-positive groups (Table S10). Pairwise PERMANOVA analysis of immune-related genes among dietary groups showed that all three diets differed significantly in immune gene expression profiles (C vs. H: R^2^ = 0.093, q = 0.009; C vs. S: R^2^ = 0.225, q = 0.001; H vs. S: R^2^ = 0.121, q = 0.002; Table S11). In contrast, pairwise comparisons between balantidia-positive and -negative fish within each dietary group showed no significant differences (C1 vs. C0: R^2^ = 0.068, q = 0.851; H1 vs. H0: R^2^ = 0.063, q = 0.851; S1 vs. S0: R^2^ = 0.143, q = 0.198; Table S12). These results indicate that balantidia colonization status was not associated with a significant shift in the expression profiles of immune-related genes. Quantitative real-time PCR was performed on four immune genes across the three comparisons to validate the RNA-seq results. The fold changes obtained by qRT-PCR were consistent with the RNA-seq expression levels, and both assays showed identical trends (Figure 6C).

## 3. Discussion

The gut microbiota is a complex microbial community that colonizes the host gut, establishing long-term symbiotic relationships with its host [24]. The interactions between the host and its microbiota are modulated by the diversity and relative abundance of intestinal microbes. These microbes not only directly participate in nutrient digestion and absorption, intestinal metabolism, and regulation of the host immune system, but also defend the host against pathogen invasion [24,25,26]. However, parasites may influence the composition of the host microbiota through various mechanisms [27,28,29]. In this study, we evaluated the effects of balantidia colonization on the hindgut microbiota, metabolites and intestinal transcriptome of grass carp under different dietary regimes. Although no obvious histological lesions were observed in the hindgut regardless of parasite load, grass carp colonized by balantidia exhibited slightly lower body length and body weight compared with balantidia-negative individuals at the end of the feeding period. Overall, no statistically significant effect of balantidia colonization on growth performance was detected under the tested conditions.

In our previous study, we found that grass carp colonized by balantidia exhibited increased bacterial diversity [5]. However, in the present study, two-way ANOVA showed a significant Diet × Status interaction for Shannon and Simpson diversity, and Chao1 and ACE richness estimates were mainly influenced by diet, with no significant colonization effect in any group. For microbial community structures, diet, balantidia status, and their interaction were all significantly associated with hindgut microbial community structure, which is consistent with previous findings of an association between balantidia colonization and intestinal microbiota composition [5]. The effect of *B. ctenopharyngodoni* colonization on the microbial community is highly context-dependent, varying with the dietary regime. This is consistent with the above alpha-diversity results, where a significant Diet × Status interaction was also detected for Shannon and Simpson diversity. Our findings suggest that dietary composition may modulate the association between *B. ctenopharyngodoni* and the hindgut microbiota, but the magnitude depended on diet. Bacteroidetes are core commensal bacteria in the fish digestive tract, possessing a large repertoire of carbohydrate-degrading enzymes to break down plant polysaccharides. The subsequent fermentation products, such as short-chain fatty acids, provide energy for intestinal epithelial cells and strengthen intestinal barrier function. In addition, Bacteroidetes are linked to detailed metabolic functions regarding nutrient digestion and immune modulation [30]. The increased relative abundance of Bacteroidetes in the C1 group is consistent with a potential enhancement of polysaccharide utilization. However, 16S rRNA data alone cannot demonstrate increased polysaccharide degradation or SCFA production, and this remains a hypothesis requiring functional validation. Previous studies have reported that high-protein diets generally reduce the relative abundance of proteobacteria in the fish intestine [31,32,33]. Balantidia colonization reshaped the bacterial relative abundance pattern in the S group, where the relative abundances of alphaproteobacteria and betaproteobacteria significantly increased instead. The association between the Firmicutes/Bacteroidetes ratio and obesity or metabolic dysfunction remains controversial and is highly context-dependent [34,35,36,37]. Thus the significantly decreased F/B ratio indicates a shift from Firmicutes-dominated to Bacteroidetes-dominated microbiota, reflecting a reshaped hindgut microbial community under balantidia colonization.

The host–parasite–microbiota interplay is a complex and dynamic tripartite interaction. On the one hand, parasites may affect the intestinal microbiota through multiple factors, but the exact mechanisms remain to be clearly understood [38]. Furthermore, microbial responses differ across parasite species, and even among distinct subtypes of the same parasite species. On the other hand, parasite-associated microbes may play multiple roles within the host intestine, ranging from behavioral manipulation and nutritional modulation to inflammatory regulation [39]. A healthy and functional intestine is both dynamic and plastic; the crosstalk between host immune and physiologic adaptation, together with external factors such as diet, microbiota, and metabolites, collectively determines host homeostasis during parasite colonization. Intestinal microbiota are closely linked to host health status through regulation of nutritional metabolism and immune homeostasis; thus, alterations in gut microbiota composition and microbial metabolites are strongly associated with metabolic disorders and their progression [40,41]. To gain functional insight into the microbial community shifts observed, metabolomic profiling was employed to capture differences in intestinal metabolites. It should be noted that the broad pathway enrichment observed reflected overall shifts in metabolic profiles and might not be direct evidence of specific physiological consequences. Diet was the dominant factor shaping the hindgut metabolomic profiles (26.0% of the variation) and microbial community structure (13.7%), while *B. ctenopharyngodoni* also had a significant main effect in both datasets, although its effect size was smaller. The Diet × Status interaction was significant in both datasets, but its effect size was larger for the microbial community (R^2^ = 0.231) than for the metabolome (R^2^ = 0.085), suggesting that the diet-dependent association with colonization may be more pronounced at the microbial community level than at the overall metabolic level. The functional profiles of hindgut microbial metabolites in grass carp colonized by balantidia were significantly enriched in pathways such as core energy metabolism, arachidonic acid metabolism, and steroid hormone metabolism. Because these metabolites may be derived from both the host and the microbiota, these findings were associations rather than direct evidence of microbial functional changes. Studies have shown that moderate dietary supplementation with arachidonic acid was beneficial for growth, development, survival, and other physiological traits in fish [42,43,44,45,46], while high levels of arachidonic acid may induce inflammatory responses, oxidative stress, and up-regulation of pro-apoptotic gene expression levels [47]. Thus, arachidonic acid and its derivatives participate in multiple physiological processes. In this study, balantidia colonization altered the metabolic profiles of the hindgut microbiota, which may further influence host intestinal health and physiological homeostasis.

Parasites may be affected, directly or indirectly, by cytokines and other immune effector molecules, and they may in turn affect the cells of the immune system [15,38,48]. For instance, some *Blastocystis* subtypes can not only support mucosal barrier integrity but also induce anti-inflammatory immune responses through microbial metabolites [49,50]. Interleukins have been demonstrated to facilitate the expulsion of parasites from the digestive tracts and the establishment of resistance to infection [51,52]. Induction of IL-10 responses can prevent a cytokine storm during protozoan infections [53]. Given the close association between parasite and host intestinal immunity, we performed transcriptome analysis on hindgut tissues to assess the immunomodulatory effects of balantidia. The transcriptomic analysis revealed considerable numbers of DEGs in each comparison, indicating that balantidia colonization was accompanied by transcriptional adjustments in general biological processes, including metabolic and catalytic activities, and cell cycle and DNA replication-related functions. Although dietary groups differed significantly in immune-related gene expression profiles, balantidia-positive and -negative fish did not differ significantly within any dietary group under the tested conditions. This finding suggested that balantidia colonization may not trigger a strong host immune or inflammatory reaction, regardless of dietary background.

## 4. Materials and Methods

### 4.1. Fish Collection and Rearing Experiment

Before the rearing experiment, a total of 41 two-summer-old grass carp (28.9 ± 2.7 cm in body length, 384.3 ± 94.1 g in body weight) were collected from a culture pond in Honghu City, Hubei Province, using a fishnet. An initial assessment, including measurements of body length, body weight, and histological observation, was performed on the sampled fish. The experimental procedures and protocols have been approved by the Animal Ethical and Welfare Committee of the Institute of Hydrobiology, Chinese Academy of Sciences (No. IHB/LL/2023036). All methods were carried out in accordance with relevant guidelines and regulations. The prevalence of balantidia in this stock was determined to be 82.9%. 10 balantidia-positive (including high-intensity balantidia and low-intensity balantidia colonization) and 5 balantidia-negative hindgut samples were fixed in 4% paraformaldehyde (Beyotime, Shanghai, China), dehydrated through a graded alcohol-xylene series, embedded in paraffin (Solarbio, Beijing, China), sectioned and stained with hematoxylin and eosin (H&E, Solarbio, Beijing, China). Histological observation was performed under a light microscope (ZEISS Axio Imager A2, Jena, Germany) equipped with a digital camera (ZEISS Axiocam 506 color, Oberkochen, Germany).

Subsequently, the fish were randomly divided into three groups (30 fish per dietary treatment) and acclimated in net cages for 7 days. The prevalence of balantidia in the stock population was determined before the feeding trial. However, individual infection status was not used as a stratification variable before random allocation to the three dietary groups. Over a two-month period, the fish were fed to apparent satiation twice daily with one of the three diets: a plant-based diet (ryegrass, *Lolium perenne* L., 10% body weight, group C), a mixed diet (ryegrass supplemented with commercial feed of Tongwei 103# (Tongwei Co., Ltd., Chengdu, China), 5% and 1.5% body weight, respectively, group H), or a commercial feed only (Tongwei 103#, 3% body weight, group S). The commercial feed contains fish meal, wheat, soybean meal, rapeseed meal, vitamin premix, and trace mineral premix. The guaranteed nutrient levels are crude protein ≥ 30%, crude fat ≥ 3%, lysine ≥ 1.5%, crude fiber ≤ 10%, and crude ash ≤ 15%.

### 4.2. Sample Collection

At the end of the rearing experiment, fish were taken out of net cages, anesthetized with 300 mg/L MS-222 (tricaine methanesulfonate, Sigma-Aldrich, St. Louis, MO, USA), and their growth parameters were recorded. Each fish was then rapidly dissected, and the hindgut was exposed and opened under aseptic conditions. The colonization status was determined individually for each fish based on the presence or absence of balantidia in the hindgut. Fish were then classified within each dietary group as balantidia-negative (0) or balantidia-positive (1), yielding six subgroups: C0, C1, H0, H1, S0, and S1. The primary comparisons were C1 vs. C0 (within the plant-based diet group), H1 vs. H0 (within the mixed diet group), and S1 vs. S0 (within the commercial feed group). Hindgut contents were collected by scraping with a sterile spatula, immediately frozen in liquid nitrogen, and stored for 16S rRNA amplicon sequencing and metabolite profiling. Additionally, a 1 cm segment of the hindgut was excised, gently rinsed with sterile PBS and stored in liquid nitrogen until RNA extraction. Each fish constituted one experimental unit, and all downstream statistical analyses were performed at the individual-fish level.

For the microbiome analysis, hindgut contents from 5 fish per subgroup (C0, C1, H0, H1, S0, S1; 30 samples in total) were subjected to 16S rRNA amplicon sequencing. The same 5 samples per subgroup were used for untargeted metabolomics. For transcriptomics, hindgut tissues from 6 fish per subgroup were sequenced. For qRT-PCR verification, 3 biological replicates per subgroup were analyzed, with three technical replicates per sample.

### 4.3. 16S rRNA Amplicon Sequencing and Data Processing

Total genomic DNA was extracted from each hindgut content sample using QIAamp^®^ DNA Stool Mini Kit (Qiagen, Hilden, Germany) according to the manufacturer’s instructions. DNA quality and quantity were assessed by 1% agarose gel electrophoresis and a NanoDrop 2000 spectrophotometer (Thermo Fisher Scientific, Wilmington, DE, USA). The V3–V4 hypervariable region of the 16S rRNA gene was amplified by polymerase chain reaction (PCR). PCR was performed with an initial denaturation step at 94 °C for 4 min, followed by 25 cycles of 94 °C for 30 s, 56 °C for 30 s and 72 °C for 60 s, then a final extension step at 72 °C for 5 min. The resulting PCR products were separated on a 2% agarose gel and purified using the QIAquick Gel Extraction Kit (Qiagen, Hilden, Germany). Sequencing libraries were constructed with the TruSeq^®^ DNA PCR-Free Sample Preparation Kit (Illumina, San Diego, CA, USA), and sequenced on an Illumina Nova 6000 platform with 2 × 150 bp paired-end reads. Approximately 200,000 raw reads were obtained per sample.

Data analysis was performed on the Metware Cloud platform (https://cloud.metware.cn). Raw reads were filtered using fastp v0.22.0 to obtain high-quality clean reads (free of adapter contamination, > 150bp in length, with an average quality score >20). The processed reads were denoised and merged into amplicon sequence variants (ASVs) using the DADA2 plugin (v1.26.0) within QIIME2 (v2023.2). Taxonomical classification was performed against the SILVA 138 database [54]. Alpha-diversity indices (Shannon, Simpson, Chao1, and ACE) were calculated using the vegan (v2.6.2) and phyloseq (v1.40.0) packages in R software (v4.2.0). Normality and homogeneity of variance were assessed using Q-Q plots and Levene’s test, respectively. Two-way ANOVA was used to test the main effects of diet (C, H, S) and balantidia status (positive, negative) as well as their interaction (Diet × Status) on each alpha-diversity index. In addition, Mann–Whitney U tests (two-sided) were used to compare alpha-diversity indices between balantidia-positive and balantidia-negative fish within each dietary group (C1 vs. C0, H1 vs. H0, and S1 vs. S0). *P*-values from multiple comparisons were adjusted using the Benjamini–Hochberg false discovery rate (FDR) procedure across all tests, and FDR-adjusted q-values < 0.05 were considered statistically significant. Differential biomarker discovery was carried out using the MicrobiotaProcess package in R. Microbial community structure was analyzed using two-way PERMANOVA on Bray–Curtis distances with diet, balantidia status, and their interaction as factors. R^2^, F, and *p* values are reported for each term. Pairwise PERMANOVA was also performed to control for confounding. *p* values were adjusted using the Benjamini–Hochberg procedure. All analyses used 999 permutations with a fixed random seed (set.seed(123)) for reproducibility.

### 4.4. Metabolite Analysis of Intestinal Contents

For each sample, 25 mg of intestinal content was thawed, homogenized, and ground at 30 Hz for 20 s. A 400 μL solution of methanol/water (7:3, *v*/*v*) was added to the ground sample, which was then shaken at 1500 rpm for 5 min. After incubation on ice for 15 min, the sample was centrifuged (Eppendorf Centrifuge 5804R, Hamburg, Germany) at 12,000 rpm for 10 min, and 300 μL of the supernatant was collected and placed at −20 °C for 30 min. The supernatant was then centrifuged again (Eppendorf Centrifuge 5804R, Hamburg, Germany) at 12,000 rpm for 3min, and 200 μL of the final supernatant was transferred for LC-MS analysis.

One aliquot was analyzed under positive ion conditions and eluted from a T3 column (Waters ACQUITY Premier HSS T3 Column 1.8 μm, 2.1 mm × 100 mm) using 0.1% formic acid in water as solvent A and 0.1% formic acid in acetonitrile as solvent B. The analytical parameters were as follows: column temperature 40 °C; flow rate 0.4 mL/min; and injection volume 4 μL. Another aliquot was analyzed under negative ion conditions using the same elution gradient as in positive mode. Data acquisition was performed in information-dependent acquisition (IDA) mode using Analyst TF 1.7.1 Software (Sciex, Concord, ON, Canada).

Raw data were converted to mzXML format using ProteoWizard software v3.0.21229. Peaks with a detection rate below 50% in any group were discarded, and metabolic identification was achieved by searching the Metware in-house database. Unsupervised principal component analysis (PCA) was performed using the prcomp function in R. Differential metabolites were determined based on variable importance in projection (VIP > 1) from the OPLS-DA model (generated using MetaboAnalystR) and FDR-adjusted q values (Benjamini–Hochberg correction, q < 0.05). Metabolomic profiles were analyzed using two-way PERMANOVA on Euclidean distances. Prior to distance calculation, metabolite intensities were log_2_-transformed and normalized by total sum scaling. R^2^, F, and *p* values are reported for each term. Pairwise PERMANOVA was performed on metabolomic profiles among dietary groups, and between balantidia-positive and -negative fish. *p* values were adjusted using the Benjamini–Hochberg procedure across the three comparisons for each factor. All analyses used 999 permutations with a fixed random seed (set.seed(123)) for reproducibility.

### 4.5. RNA Extraction, Library Construction, Sequencing and Analysis

Total RNA was extracted from each hindgut sample using TRIzol Reagent (Invitrogen, Carlsbad, CA, USA) according to the manufacturer’s protocol. RNA quality and quantity were assessed using an Agilent 2100 Bioanalyzer (Agilent Technologies, Santa Clara, CA, USA). Qualified RNA was enriched with Oligo(dT)-attached magnetic beads, followed by fragmentation, reverse transcription, and end repair. First-strand cDNA was synthesized with fragmented mRNA as template and random hexamers as primers, followed by second-strand synthesis with addition of PCR buffer, dNTPs, RNase H and DNA polymerase I; then adenosine was added to the end and ligated to adapters. The resulting products were selected using AMPure XP beads, and the sequencing library was constructed after PCR amplification. The PCR conditions were set as follows: initial denaturation at 98 °C for 30 s; followed by 12 cycles of 98 °C for 10 s, 60 °C for 30 s, and 72 °C for 30 s; and a final extension at 72 °C for 5 min. Sequencing was performed on an Illumina Novaseq 6000 platform by Biomarker Technology Co., Ltd. (Beijing, China).

Raw RNA-seq data were filtered to remove low-quality reads, those containing adapter sequences, >10% unknown N bases, or >50% of bases with a low quality score ≤ 20. High-quality clean reads were aligned to the grass carp reference genome (GCA_019924925.1; Wu et al. [55]) using HISAT2 software v2.2.1 [56]. The aligned reads were subsequently quantified with featureCounts [57]. Transcriptomic profiles were analyzed using two-way PERMANOVA on Euclidean distances. Count data were filtered to retain genes with ≥10 counts in at least six samples. The model was fitted using the adonis2 function in vegan (v2.6.2) with 999 permutations. R^2^, F, and *p* values are reported for each term. To specifically examine immune-related genes, we performed pairwise PERMANOVA on a set of immune-related genes. *p* values were adjusted using the Benjamini–Hochberg procedure across the three comparisons for each factor. All analyses used 999 permutations with a fixed random seed (set.seed(123)) for reproducibility. Differential expression analysis was performed using DESeq2 with genes satisfying an FDR-adjusted q < 0.05 and |log_2_(fold change)| > 1 considered differentially expressed genes (DEGs). Heatmaps illustrating gene expression patterns were generated using the pheatmap 1.0.12 package in R. Functional annotation and classification were performed using Blast2GO [58], and KEGG orthology assignments were obtained via the KEGG Automatic Annotation Server (KAAS) [59].

### 4.6. Verification of Immune-Related Genes

To validate the RNA-seq results, a subset of immune-related genes was selected for quantitative real-time PCR (qRT-PCR). Four pairs of gene-specific primers were designed using Primer Premier 6, with β-actin as the reference gene (Table S13). The qRT-PCR was performed on a CFX connect real-time PCR detection system (BIO-RAD, CA, USA) in triplicate under the following conditions: initial denaturation at 94 °C for 3 min, followed by 34 cycles of 94 °C for 30s, 56 °C for 40s, and 72 °C for 40s, with a final extension step of 72 °C for 5 min. Fluorescence signals were captured at the end of each extension step. The 20 μL reaction mixture consisted of 10 μL of SYBR Green Real-time PCR master mix (Thermo Fisher Scientific, Shanghai, China), 1 μL of cDNA template, 1 μL of each primer, and 7 μL of nuclease-free water. Relative expression levels were calculated using the 2^−∆∆Ct^ method [60].

### 4.7. Correlation Analysis of Group-Specific Differential Microbes and Metabolites

Correlation analyses between group-specific differential microbes and metabolites were performed to explore their potential statistical associations. Discriminant microbial taxa were identified using Linear Discriminant Analysis Effect Size (LEfSe) analysis. Subsequently, associations between features were evaluated using Spearman correlation coefficients. Only correlations with a coefficient > 0.8 and a *p*-value < 0.05 were retained for network visualization using the ggraph package (version 2.2.2) in R.

## 5. Conclusions

*B. ctenopharyngodoni* has been reported to become pathogenic under predisposing conditions, often accompanied by the presence of pathogenic bacteria and intestinal inflammation. In this study, we conducted an integrated analysis combining microbial amplicon sequencing, metabolomics, and intestinal transcriptomics to comprehensively evaluate the pathogenicity of *B. ctenopharyngodoni* under different dietary regimes. Balantidia colonization may be associated with numerically higher alpha diversity of the hindgut microbiota, a reduced F/B ratio, and alterations in specific bacterial taxa and metabolites. Unlike virulent pathogens that disrupt intestinal homeostasis and provoke robust immune responses, this ciliate only mildly reshapes the hindgut microbial community structure without inducing a marked transcriptional immune response. These findings indicate that balantidia has limited adverse impacts on host health under the tested conditions and is unlikely to act as a primary pathogen. It should be noted that fish were allocated blindly from a naturally infected stock, and infection status was determined only at the end of the experiment; these findings should be interpreted as associational. Thus, experimental infection trials may further be required to determine whether it is intrinsically non-pathogenic. Given its high prevalence in grass carp, it may co-exist when intestinal disorders occur. Therefore, we should avoid simply regarding balantidia as the primary cause of intestinal diseases in grass carp when detected in diseased fish.

## Figures and Tables

**Table 1:** The detailed information on alpha diversity in all groups.

Index		C0	C1	H0	H1	S0	S1
Shannon	Mean ± SD	1.51 ± 0.41	1.99 ± 0.29	1.00 ± 0.33	1.98 ± 0.38	1.91 ± 0.54	1.42 ± 0.51
	*p* value	0.117		0.008		0.238	
	q value (BH)	0.176		0.024		0.238	
	Significance	ns		*		ns	
Simpson	Average	0.64 ± 0.18	0.79 ± 0.09	0.49 ± 0.18	0.80 ± 0.07	0.75 ± 0.10	0.55 ± 0.19
	*p* value	0.117		0.008		0.056	
	q value (BH)	0.117		0.024		0.084	
	Significance	ns		*		ns	
Chao1	Average	24.64 ± 9.45	33.20 ± 5.5	35.02 ± 7.67	38.00 ± 12.31	51.87 ± 12.98	39.00 ± 13.06
	*p* value	0.238		1.000		0.238	
	q value (BH)	0.357		1.000		0.357	
	Significance	ns		ns		ns	
ACE	Average	24.95 ± 9.83	33.46 ± 5.33	35.51 ± 7.87	38.31 ± 12.16	52.15 ± 12.76	34.67 ± 16.26
	*p* value	0.238		1.000		0.190	
	q value (BH)	0.357		1.000		0.357	
	Significance	ns		ns		ns	

Note: ns indicates balantidia-positive and balantidia-negative comparisons are not significant (q > 0.05), * indicates the comparisons are significant (q < 0.05).

## Data Availability

Raw sequences generated in this study were deposited in NGDC under Bioproject accession number PRJCA068350. Other relevant data will be made available on request.

## Supplementary Materials

The following supporting information can be downloaded at: https://www.mdpi.com/article/10.3390/ijms27219425/s1.
