# Supplementary material for: Integrated Multi-Omics Analysis Provides No Evidence for Pathogenicity of Balantidium Ctenopharyngodoni in Grass Carp Hindgut
Source: Int J Mol Sci. 2026 Oct 22;27(21):9425. doi: 10.3390/ijms27219425 (PMC13762522; doi:10.3390/ijms27219425)
Supplement: Supplementary file 1 [file ijms-27-09425-s001.zip › Supplementary Figures.pdf]

Supplementary Figures

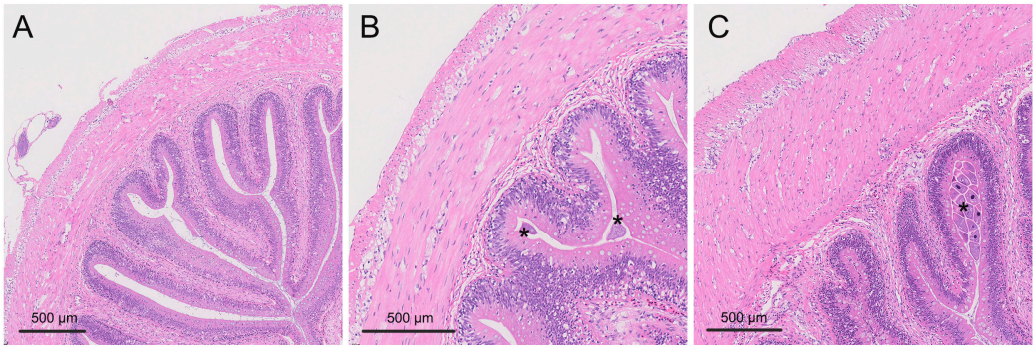

Figure S1. Histological observation of grass carp hindgut by H&E staining prior to the rearing experiment. (A) Hindgut without balantidia colonization. (B) Hindgut with low-intensity balantidia colonization (0-1,000 individuals/fish). (C) Hindgut with high-intensity balantidia colonization (> 1,000 individuals/fish). Asterisk indicates balantidia inhabited between the mucosal folds.

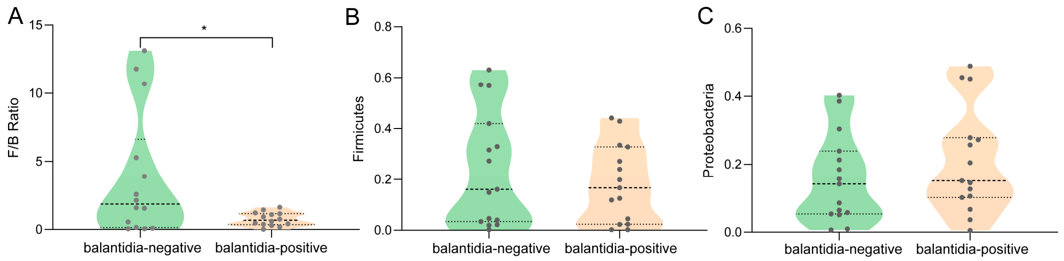

Figure S2. Effects of balantidia colonization on the Firmicutes/Bacteroidetes (F/B) ratio (A), and relative abundance of Firmicutes (B) and Proteobacteria (C). Statistical significance between groups was marked by asterisk ($P < 0.05$).

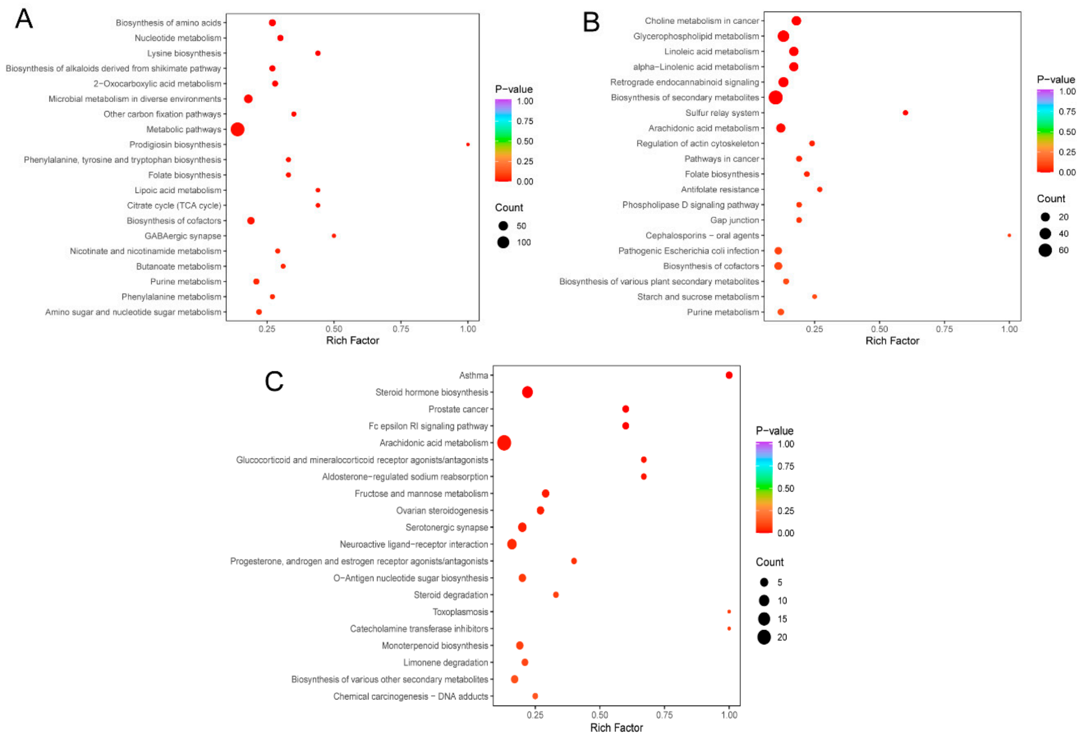

Figure S3. KEGG pathway enrichment analysis of all DAMs in the three comparisons: C1 vs. C0 (A), H1 vs. H0 (B), and S1 vs. S0 (C). Groups: C, plant-based diet (ryegrass); H, mixed diet (ryegrass + commercial feed); S, commercial feed only. “0” indicates balantidia-negative fish; “1” indicates balantidia-positive fish.

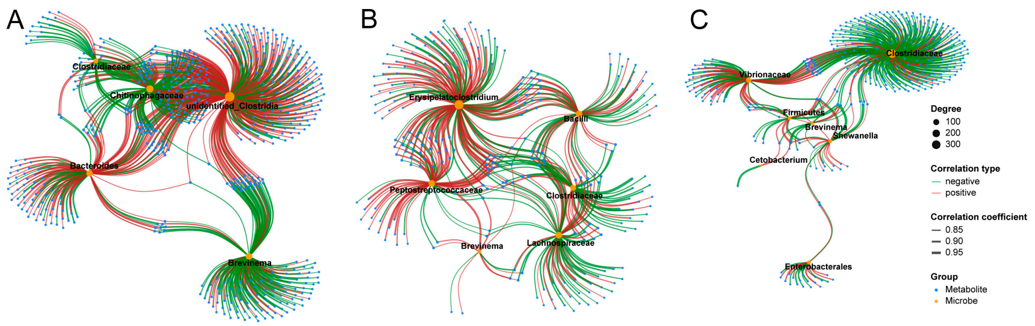

Figure S4. Correlation networks between discriminant microbial taxa and differential metabolites across groups: C1 vs. C0 (A), H1 vs. H0 (B), and S1 vs. S0 (C). Nodes represent metabolites (blue) or microbial taxa (yellow). Degree represents node connectivity. Lines indicate correlation types: red for positive and green for negative. Line thickness corresponds to correlation coefficients (ranging from 0.85 to 0.95).

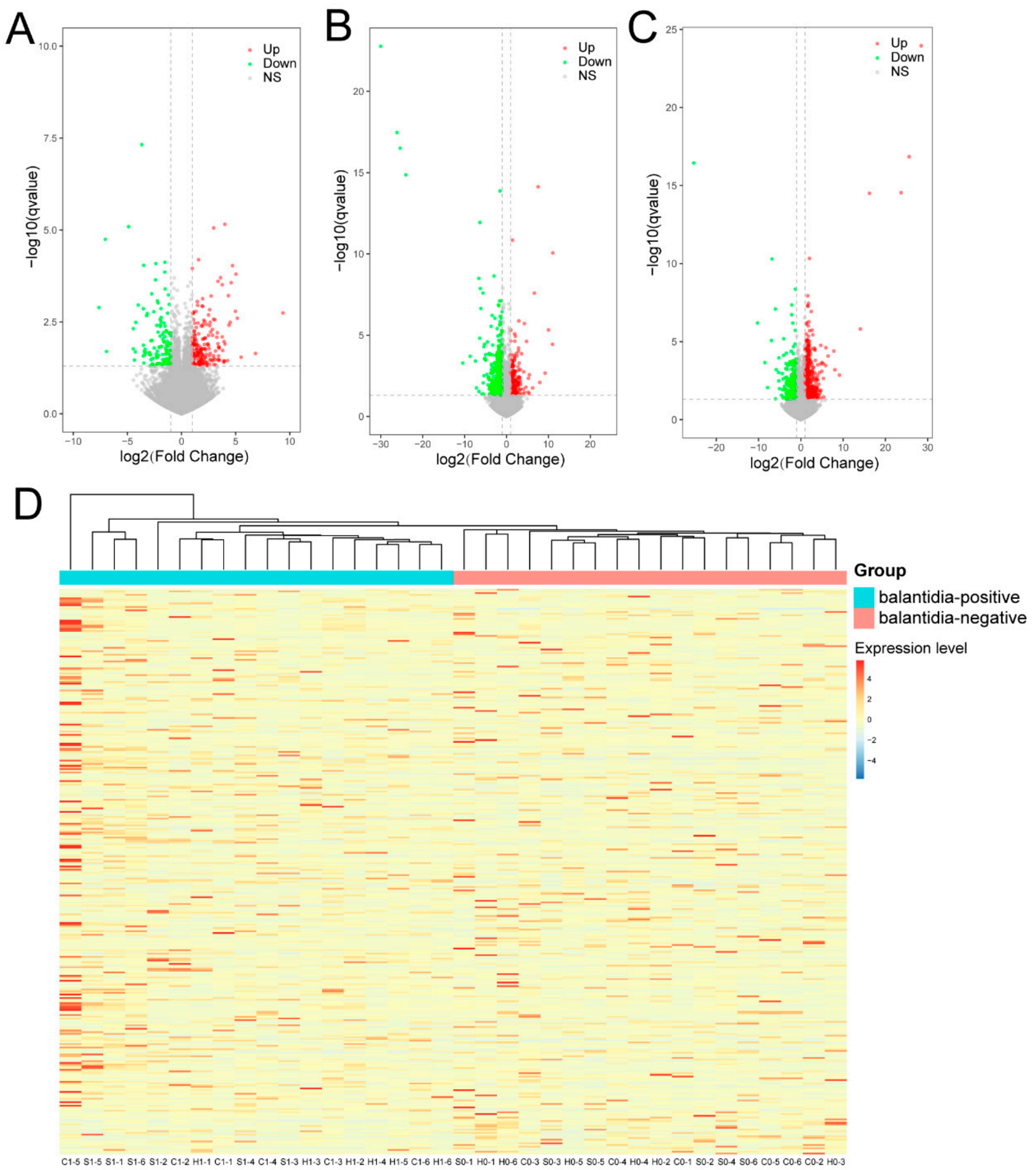

Figure S5. Volcano plots of differentially expressed genes (DEGs) for C1 vs. C0 (A), H1 vs. H0 (B), and S1 vs. S0 (C). The log_2(Fold Change) indicates the mean expression level for each gene. Hierarchical clustering heatmap of all DEGs across samples. Groups: C, plant-based diet (ryegrass); H, mixed diet (ryegrass + commercial feed); S, commercial feed only. "0" indicates balantidia-negative fish; "1" indicates balantidia-positive fish.
