# Supplementary material for: Integrated Multi-Omics Analysis Provides No Evidence for Pathogenicity of Balantidium Ctenopharyngodoni in Grass Carp Hindgut
Source: Int J Mol Sci. 2026 Oct 22;27(21):9425. doi: 10.3390/ijms27219425 (PMC13762522; doi:10.3390/ijms27219425)
Supplement: Supplementary file 1 [file ijms-27-09425-s001.zip › Supplementary Tables.pdf]

Supplementary Tables

Table S1. Statistics of microbial data of each sample.

Sample ID	Diet	Status	Raw reads	Clean reads	% Clean reads	% GC
C0_1	plant-based	negative	205336	204388	99.5	51.91
C0_2	plant-based	negative	212516	211750	99.6	52.93
C0_3	plant-based	negative	206668	206030	99.7	52.96
C0_4	plant-based	negative	207174	206616	99.7	53.39
C0_5	plant-based	negative	221036	220308	99.7	52.95
C1_1	plant-based	positive	205530	204442	99.5	48.54
C1_2	plant-based	positive	209310	208736	99.7	50.50
C1_3	plant-based	positive	237054	236484	99.8	50.67
C1_4	plant-based	positive	221182	220644	99.8	51.75
C1_5	plant-based	positive	208878	208324	99.7	51.67
H0_1	mixed	negative	235140	234470	99.7	51.25
H0_2	mixed	negative	235764	235098	99.7	48.43
H0_3	mixed	negative	208642	207844	99.6	52.83
H0_4	mixed	negative	211142	210260	99.6	48.65
H0_5	mixed	negative	213564	212928	99.7	52.68
H1_1	mixed	positive	211844	211164	99.7	50.82
H1_2	mixed	positive	218584	217946	99.7	52.40
H1_3	mixed	positive	208278	207624	99.7	51.11
H1_4	mixed	positive	210470	209886	99.7	49.78
H1_5	mixed	positive	213378	212750	99.7	49.06
S0_1	commercial	negative	209516	208966	99.7	51.94
S0_2	commercial	negative	212842	212090	99.6	50.14
S0_3	commercial	negative	236344	235638	99.7	50.08
S0_4	commercial	negative	191694	190910	99.6	50.63
S0_5	commercial	negative	237830	237184	99.7	52.86
S1_1	commercial	positive	204574	203792	99.6	50.67
S1_2	commercial	positive	206950	204848	99.0	52.57
S1_3	commercial	positive	204170	203432	99.6	51.05
S1_4	commercial	positive	207818	206994	99.6	52.02
S1_5	commercial	positive	206012	205076	99.5	52.09
Total	-	-	6419240	6396622	99.6	51.27

Note: -, not applicable.

Table S2. The effects of diet, balantidia status, and their interaction on alpha-diversity indices based on two-way ANOVA.

Index	Effect	df	*P* value	q value (BH)	Significance
	Diet	2	0.384	0.512	ns
Shannon	Status	1	0.045	0.106	ns
	Diet×Status	2	0.002	0.014	*
	Diet	2	0.477	0.572	ns
Simpson	Status	1	0.124	0.186	ns
	Diet×Status	2	0.002	0.014	*
	Diet	2	0.007	0.028	*
Chao1	Status	1	0.91	0.91	ns
	Diet×Status	2	0.083	0.142	ns
	Diet	2	0.014	0.042	*
ACE	Status	1	0.874	0.91	ns
	Diet×Status	2	0.053	0.106	ns

Note: *q < 0.05; ns, not significant.

Table S3. Two-way PERMANOVA of microbial community composition testing the effects of diet, status, and their interaction.

Factor	df	R^2	F	*P* value	Significance
Diet	2	0.137	2.962	0.001	***
Status	1	0.076	3.265	0.008	**
Diet×Status	2	0.231	4.971	0.001	***

Note: ** $P < 0.01$; *** $P < 0.001$.

Table S4. Pairwise PERMANOVA of microbial community composition among dietary groups.

Comparison	R^2	F	*P* value	q value (BH)	Significance
C vs H	0.072	1.403	0.06	0.054	ns
C vs S	0.166	3.573	0.009	0.006	**
H vs S	0.078	1.518	0.151	0.129	ns

Note: **q < 0.01; ns, not significant.

Table S5. Pairwise PERMANOVA of microbial community composition between balantidia-positive and -negative fish.

Comparison	R^2	F	*P* value	q value (BH)	Significance
C1 vs C0	0.481	7.409	0.01	0.030	*
H1 vs H0	0.203	2.042	0.034	0.034	*
S1 vs S0	0.389	5.095	0.02	0.030	*

Note: *q < 0.05.

Table S6. Two-way PERMANOVA of metabolomic profiles testing the effects of diet, status, and their interaction.

Factor	df	R^2	F	*P* value	Significance
Diet	2	0.260	5.287	0.001	***
Status	1	0.064	2.596	0.007	**
Diet×Status	2	0.085	1.721	0.031	*

Note: *$P < 0.05$; **$P < 0.01$; ***$P < 0.001$.

Table S7. Pairwise PERMANOVA of metabolomic profiles among dietary groups.

Comparison	R^2	F	*P* value	q value (BH)	Significance
C vs H	0.165	3.558	0.001	0.001	***
C vs S	0.314	8.242	0.001	0.001	***
H vs S	0.135	2.805	0.001	0.001	***

Note: ***q < 0.001.

Table S8. Pairwise PERMANOVA of metabolomic profiles between balantidia-positive and -negative fish.

Comparison	R^2	F	*P* value	q value (BH)	Significance
C1 vs C0	0.249	2.647	0.021	0.039	*
H1 vs H0	0.169	1.628	0.080	0.080	ns
S1 vs S0	0.190	1.875	0.026	0.039	*

Note: *q < 0.05; ns, not significant.

Table S9. Two-way PERMANOVA of transcriptomic profiles testing the effects of diet, status, and their interaction.

Factor	df	R^2	F	*P* value	Significance
Diet	2	0.161	3.202	0.001	***
Status	1	0.032	1.267	0.131	ns
Diet×Status	2	0.053	1.054	0.316	ns

Note: ****P* < 0.001; ns, not significant.

Table S10. List of genes involved in immune responses

Gene ID	Gene name	C1 vs. C0				H1 vs. H0				S1 vs. S0			
		log2FC	*P* value	q value	significance	log2FC	*P* value	q value	significance	log2FC	*P* value	q value	significance
c4	Complement component C4	-0.214	0.485	0.966	ns	0.036	0.874	0.979	ns	-0.255	0.364	0.702	ns
c5	Complement component C5	0.467	0.461	0.966	ns	-0.342	0.526	0.913	ns	-1.473	0.001	0.028	down
c6	Complement component C6	0.091	0.640	0.966	ns	0.102	0.712	0.913	ns	0.031	0.900	0.953	ns
c9	Complement component C9	-0.524	0.828	0.966	ns	0.013	0.991	0.997	ns	1.817	0.164	0.501	ns
LOC127495323	α-2-macroglobulin	0.472	0.412	0.966	ns	0.709	0.451	0.913	ns	0.239	0.769	0.897	ns
irf6	Interferon regulatory factor 6	-0.045	0.651	0.966	ns	0.079	0.473	0.913	ns	0.034	0.714	0.869	ns
irf3	Interferon regulatory factor 3	0.412	0.202	0.844	ns	0.471	0.053	0.913	ns	0.297	0.339	0.702	ns
irf7	Interferon regulatory factor 7	1.970	0.025	0.844	ns	0.349	0.407	0.913	ns	0.664	0.343	0.702	ns
irf8	Interferon regulatory factor 8	-0.166	0.391	0.966	ns	-0.092	0.656	0.913	ns	-0.185	0.260	0.607	ns
irf9	Interferon regulatory factor 9	0.280	0.193	0.844	ns	0.072	0.403	0.913	ns	-0.115	0.390	0.705	ns
il1b	Interleukin-1 beta	0.342	0.473	0.966	ns	-0.246	0.573	0.913	ns	0.837	0.121	0.473	ns
ifitm1	Interferon-induced transmembrane protein 1	-0.400	0.060	0.844	ns	0.016	0.943	0.997	ns	0.330	0.207	0.552	ns
il21	Interleukin-21	-0.056	0.807	0.966	ns	-0.376	0.258	0.913	ns	0.050	0.828	0.927	ns
il10	Interleukin-10	0.604	0.126	0.844	ns	-0.001	0.997	0.997	ns	0.774	0.008	0.084	ns
il13	Interleukin-13	0.265	0.872	0.977	ns	1.103	0.296	0.913	ns	-1.136	0.484	0.753	ns
il15	Interleukin-15	-0.029	0.847	0.968	ns	-0.159	0.184	0.913	ns	-0.098	0.534	0.779	ns
il16	Interleukin-16	-0.006	0.950	0.985	ns	-0.026	0.846	0.976	ns	-0.015	0.918	0.953	ns
il17d	Interleukin-17D	-0.116	0.671	0.966	ns	0.042	0.913	0.997	ns	0.252	0.580	0.779	ns
il17a/f3	Interleukin-17A/F3	-0.339	0.286	0.893	ns	-0.399	0.134	0.913	ns	0.159	0.689	0.857	ns
il4	Interleukin-4	-0.469	0.428	0.966	ns	0.699	0.283	0.913	ns	-1.149	0.034	0.272	ns
il6	Interleukin-6	-0.180	0.120	0.844	ns	-0.094	0.580	0.913	ns	-0.008	0.971	0.989	ns

Table S10 (Continued)

Gene ID	Gene name	C1 vs. C0				H1 vs. H0				S1 vs. S0			
		log2FC	*P* value	q value	significance	log2FC	*P* value	q value	significance	log2FC	*P* value	q value	significance
il34	Interleukin-34	0.049	0.801	0.966	ns	-0.075	0.701	0.913	ns	-0.172	0.356	0.702	ns
tnfb	tumor necrosis factor-beta	0.273	0.254	0.889	ns	0.104	0.638	0.913	ns	0.664	0.001	0.028	ns
tgfb2	transforming growth factor beta 2	0.195	0.225	0.844	ns	0.172	0.379	0.913	ns	-0.053	0.815	0.927	ns
LOC127498357	T cell receptor beta chain	-0.002	0.985	0.985	ns	-0.179	0.314	0.913	ns	0.159	0.376	0.702	ns
cd79a	CD79A	-0.156	0.645	0.966	ns	-0.055	0.854	0.976	ns	-0.259	0.568	0.779	ns
nitr9	Novel immune-type receptor 9	-0.248	0.636	0.966	ns	0.454	0.332	0.913	ns	-1.015	0.170	0.501	ns
cd180	CD180 molecule	0.377	0.164	0.844	ns	0.315	0.231	0.913	ns	0.112	0.628	0.818	ns
LOC127513741	CD209 antigen-like	-0.449	0.306	0.893	ns	0.347	0.113	0.913	ns	-0.030	0.917	0.953	ns
LOC127497641	Lymphocyte activation gene 3 protein-like	0.873	0.065	0.844	ns	0.259	0.376	0.913	ns	0.354	0.257	0.607	ns
psme2	proteasome activator complex subunit 2	0.144	0.319	0.893	ns	0.071	0.737	0.913	ns	0.346	0.107	0.473	ns
cd36	CD36 antigen	-2.314	0.193	0.844	ns	-0.606	0.750	0.913	ns	-2.350	0.133	0.473	ns
itln1	Intelectin 1	-0.104	0.940	0.985	ns	-0.933	0.518	0.913	ns	-2.043	0.129	0.473	ns
nktr	natural killer cell triggering receptor	0.180	0.296	0.893	ns	-0.149	0.437	0.913	ns	-0.084	0.644	0.820	ns
tlr22	Toll-like receptor 22	-0.094	0.643	0.966	ns	0.109	0.700	0.913	ns	-0.292	0.145	0.478	ns
tlr2	Toll-like receptor 2	-0.082	0.591	0.966	ns	-0.124	0.582	0.913	ns	-0.256	0.094	0.473	ns
tlr3	Toll-like receptor 3	0.390	0.218	0.844	ns	0.100	0.720	0.913	ns	0.149	0.455	0.728	ns
tlr9	Toll-like receptor 9	0.138	0.730	0.966	ns	-0.205	0.739	0.913	ns	0.294	0.408	0.714	ns
tlr7	Toll-like receptor 7	0.453	0.186	0.844	ns	0.153	0.716	0.913	ns	0.356	0.324	0.702	ns

Table S10 (Continued)

Gene ID	Gene name	C1 vs. C0				H1 vs. H0				S1 vs. S0			
		log2FC	*P* value	q value	significance	log2FC	*P* value	q value	significance	log2FC	*P* value	q value	significance
tlr1	Toll-like receptor 1	-0.141	0.497	0.966	ns	0.135	0.378	0.913	ns	-0.302	0.118	0.473	ns
tlr21	Toll-like receptor 21	0.513	0.395	0.966	ns	1.146	0.001	0.056	ns	0.315	0.515	0.779	ns
cldn1	Claudin 1	-0.803	0.790	0.966	ns	0.046	0.984	0.997	ns	1.693	0.584	0.779	ns
cldn2	Claudin 2	0.469	0.636	0.966	ns	1.529	0.087	0.913	ns	-0.119	0.919	0.953	ns
LOC127495378	Claudin 4	0.202	0.713	0.966	ns	0.224	0.599	0.913	ns	-0.418	0.450	0.728	ns
rcn2	Reticulocalbin 2	0.022	0.932	0.985	ns	-0.154	0.518	0.913	ns	-0.053	0.741	0.883	ns
card11	Caspase recruitment domain family, member 11	0.062	0.596	0.966	ns	-0.070	0.634	0.913	ns	0.001	0.992	0.992	ns
card9	Caspase recruitment domain family, member 9	-0.248	0.226	0.844	ns	-0.102	0.676	0.913	ns	0.146	0.580	0.779	ns
casp7	Caspase-7	0.292	0.073	0.844	ns	0.098	0.629	0.913	ns	0.259	0.076	0.473	ns
pxmp4	Peroxisomal membrane protein 4	0.053	0.794	0.966	ns	0.196	0.252	0.913	ns	0.187	0.426	0.723	ns
pxmp2	Peroxisomal membrane protein 2	0.066	0.791	0.966	ns	0.146	0.520	0.913	ns	0.823	0.003	0.056	ns
gss	Glutathione synthetase	0.098	0.605	0.966	ns	-0.241	0.193	0.913	ns	0.167	0.182	0.510	ns
srm	Spermidine synthase	-0.036	0.828	0.966	ns	0.090	0.588	0.913	ns	0.430	0.080	0.473	ns
cat	Catalase	0.016	0.937	0.985	ns	-0.035	0.813	0.969	ns	-0.167	0.257	0.607	ns
ggcta	gamma-glutamylcyclotransferase	-0.008	0.977	0.985	ns	-0.011	0.966	0.997	ns	0.692	0.009	0.084	ns

Note: ns indicates not significant; down indicates down-regulated.

Table S11. Pairwise PERMANOVA of immune-related gene expression profiles among dietary groups.

Comparison	R^2	F	*P* value	q value (BH)	Significance
C vs H	0.093	2.260	0.009	0.009	**
C vs S	0.225	6.403	0.001	0.001	***
H vs S	0.121	3.035	0.005	0.002	**

Note: **q < 0.01; ***q < 0.001.

Table S12. Pairwise PERMANOVA of immune-related gene expression profiles between balantidia-positive and -negative fish.

Comparison	R^2	F	*P* value	q value (BH)	Significance
C1 vs C0	0.068	0.732	0.824	0.851	ns
H1 vs H0	0.063	0.671	0.851	0.851	ns
S1 vs S0	0.143	1.664	0.066	0.198	ns

Note: ns, not significant.

Table S13. Quantitative real-time PCR primers used in this study.

Gene name	Forward primer (5'-3')	Reverse primer (5'-3')
β-Actin	GGCTGTGCTGTCCCTGTA	GGGCATAACCCTCGTAGAT
IL-10	GGGCTTTCCTGTGAGGCTGAA	GCTGTTGGCAGAATGGTCTCC
IL-17A/F3	AGGACAAGCCGTGGTTCATCAT	GCCTCAGAGATTTGCACTGGGA
IL-17D	TCGCTCTTGTCTGGACTTACCG	CGCTAGACGAGACGCTGATTGA
TGF-β2	TACATTGACAGCAAGGTGGTG	TCTTGTTGGGGATGATGTAATT
